# Profiling and characterization of SARS-CoV-2 mutants’ infectivity and antigenicity

**DOI:** 10.1038/s41392-020-00302-8

**Published:** 2020-09-03

**Authors:** Lin Wang, Ling Wang, Hui Zhuang

**Affiliations:** Department of Microbiology and Infectious Disease Center, School of Basic Medical Sciences, Peking University Health Science Center, Beijing, China

**Keywords:** Infectious diseases, Microbiology

**Fig. 1:**
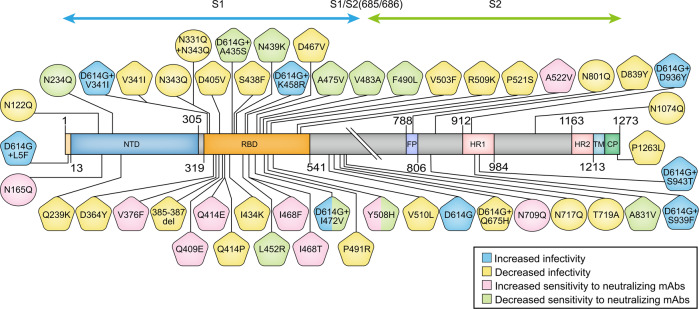
Schematic illustration of amino acid changes in spike protein. Mutations yielding at least fourfold changes in infectivity and neutralizing reactivity to mAbs are shown in the figure

Using high-throughput pseudovirus assay in conjunction with neutralizing antibodies, Wang et al. investigated 80 natural variants and 26 glycosylation-spike (S) mutants of SARS-CoV-2 in terms of infectivity and antigenicity.^1^ They identified several mutations that can critically affect the virus infectivity and reactivity to neutralizing antibodies (Fig. 1). This study provides abundant and comprehensive data that will help us better understand the impact of mutations in S protein, and may contribute to future design of potent vaccine candidate and monoclonal antibodies (mAbs).

SARS-CoV-2, the causative agent of the widespread COVID-19 pandemic, has caused at least 17.2 million infections and 671,000 deaths worldwide by the end of July 2020 (https://coronavirus.jhu.edu/). Currently, there is an urgent need for specific antiviral therapy and vaccine against SARS-CoV-2. The S protein is the key element of SARS-CoV-2 that determines the viral infectivity and induces host protective immune response.^2^ However, the rapid global spread provides the virus with an opportunity for natural selection, and several naturally occurring mutations in S protein have been identified. Given the crucial role of S protein in virus infection/transmission and vaccine/mAbs development, the authors investigated the biological significance of natural variants with amino acid change(s) and mutants at the putative N-linked glycosylation sites in the S protein.

Previously, the authors have established a sophisticated pseudovirus assay system and constructed a series of pseudoviruses of emerging and re-emerging viruses, including MERS-CoV, rabies virus, Ebola virus, Marburg virus, Lassa virus, Chikungunya virus, Nipah virus, Rift valley virus, and others.^3,4^ In this study, 106 pseudotyped viruses of SARS-CoV-2 were constructed to infect a series of human and animal cell lines, and the result demonstrated that the D614G variants or combined variants such as D614G + V341I, D614G + K458R, D614G + I472V, D614G + D936Y, D614G + S939F, and D614G + S943T showed 4- to 100-fold increased infectivity compared to the reference Wuhan-1 strain (GenBank: MN908947). Indeed, increasing the frequency and global dominance of the D614G variant has been seen, and the increase may suggest a fitness advantage of this variant. Potential association of D614G with higher viral loads in COVID-19 patients has been observed, but the link between the variant and disease severity is yet to be clarified.^5^ Furthermore, the increased infectivity and possibly increased production in the cell culture system of certain variants observed in this study may suggest a potential for development of the inactivated vaccine seed strain in the future.

In addition to amino acid changes in S protein of SARS-CoV-2, the authors also investigated the impact of deletions of 22 putative glycosylation sites. Six glycosylation mutants were determined as of low infectivity. The ablation of both, but not either, N331 and N343 glycosylation at the receptor-binding domain (RBD) drastically reduced infectivity. The results indicated that the glycosylation of S protein may play an important role in the enhancement of SARS-CoV-2 infection. In the aspect of vaccine development, strains containing glycosylation site mutation should be discreetly evaluated before being designated as a seed strain. Moreover, the impact of different expression systems on S-protein glycosylation site should also be taken into account. It is necessary to monitor the mutation of glycosylation sites in circulating SARS-CoV-2 strains to further assess the potential impact in terms of virus transmission and disease severity.

Subsequently, the author managed to investigate the antigenicity of the infectious mutants using 13 neutralizing mAbs. Notably, they discovered ten mutations, such as N234Q, L452R, A475V, V483A, and F490L, which were remarkably resistant to some mAbs. Most of the mutations located in the RBD. By analyzing the epitope of three well-characterized mAbs, P2B-2F6, CB6, and H014 on the trimer and RBD, the authors discovered that several variants that are resistant to these mAbs sit in the binding epitope of RBD. The results of the neutralizing assay using human convalescent sera were mostly consistent with mAbs, and the differences of neutralization activity, though less than fourfold, were statistically significant. This part of the study is of great importance for vaccine and mAb therapy development as it provides evidence of how circulating natural variants react to neutralizing antibodies. Desirable vaccine candidates or mAbs should have broad-spectrum reactivity to neutralize all circulating variants. Also, development and design of cocktail therapy combining two or more mAbs that can target all mutations may be of particular interest in the future.

In summary, Wang and colleagues thoroughly investigated the infectivity and antigenicity of pseudotyped SARS-CoV-2 with different mutations in S protein. The data are representative as the authors evaluated an abundant number of S-protein variants that are circulating worldwide. Several amino acid changes in S protein could critically affect the virus infectivity and the reactivity to mAbs. Furthermore, the use of high-throughput pseudovirus assay is indeed promising as it can rapidly assess the potential impact of circulation mutations on the vaccine or mAb-neutralizing reactivity in biosafety level-2 environment. Future development of vaccine and mAbs should be carefully assessed for their protective or inhibitory effect on emerging widespread mutations.
